# Comparison among bright green fluorescent proteins in *C. elegans*

**DOI:** 10.17912/micropub.biology.001447

**Published:** 2025-01-18

**Authors:** Sydney Ko, Kota Mizumoto

**Affiliations:** 1 Graduate Program in Cell and Developmental Biology, Life Sciences Institute, University of British Columbia, Vancouver, British Columbia, Canada; 2 Department of Zoology, Life Sciences Institute, Djavad Mowafaghian Centre for Brain Health, University of British Columbia, Vancouver, British Columbia, Canada

## Abstract

Green fluorescent proteins (GFPs) are invaluable tools for visualizing cells and proteins across model systems. Efforts have been made to generate brighter fluorescent proteins such as eGFP, GFPnovo2, mNeonGreen, and mStayGold. Here, we generated single-copy knock-in *C. elegans* strains for these GFP variants and directly compared their brightness and photostability. We confirmed that mStayGold is brighter and more photostable than eGFP, GFPnovo2, and mNeonGreen, suggesting that it may hold advantages over other GFP variants in experiments where brightness and photostability are important factors.

**Figure 1. Comparison among green fluorescent proteins in C. elegans:**
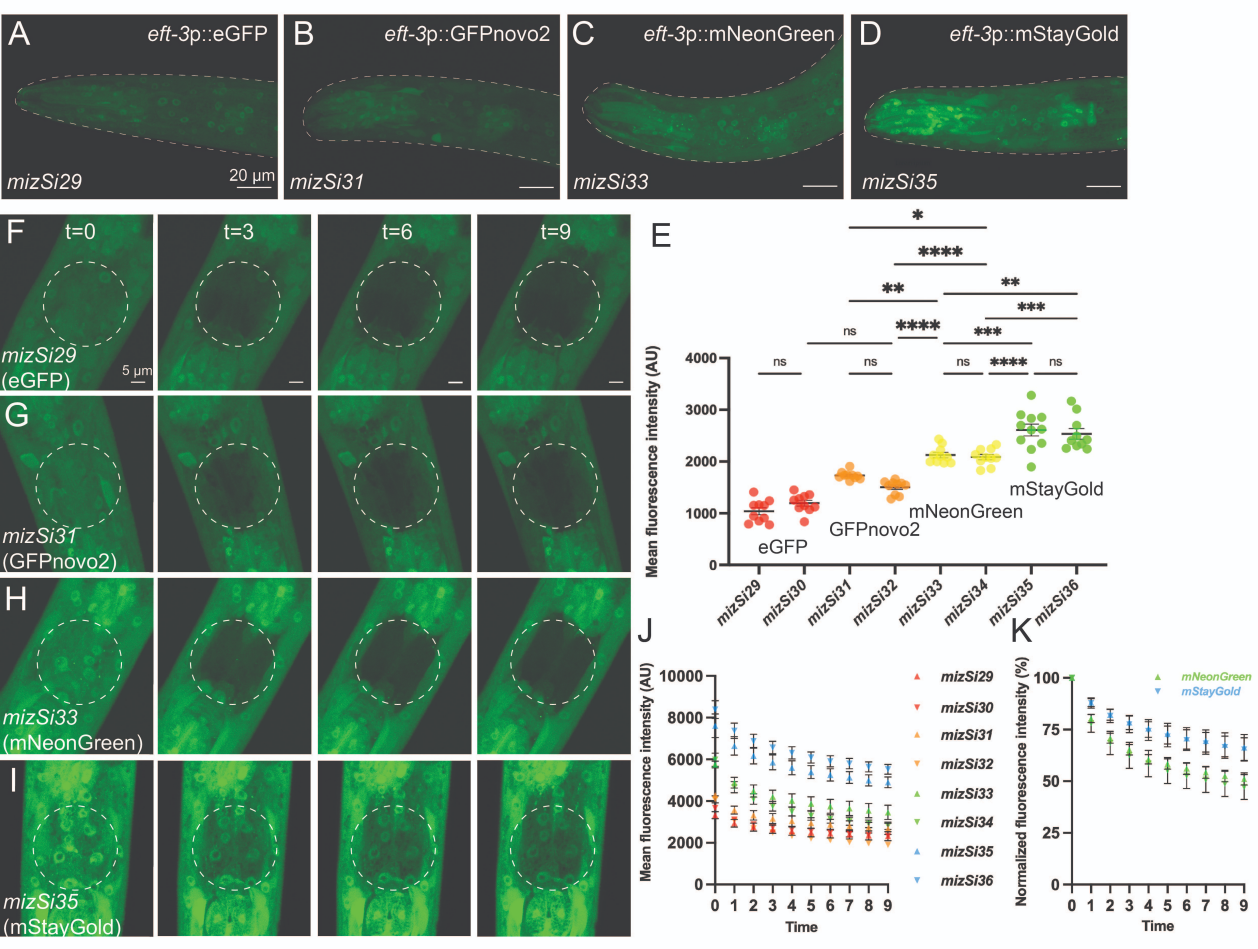
(A-D) Representative images of the head of L4 animals expressing GFP variants, (A) *eft-3* p::eGFP ( *mizSi29* ), (B) *eft-3* p::GFPnovo2 ( *mizSi31* ), (C) *eft-3* p::mNeonGreen ( *mizSi33* ), and (D) *eft-3* p::mStayGold ( *mizS35* ). Dashed outlines indicate the region of interest used for quantification. Scale bar: 20 μm (E) Quantifications of the mean fluorescence intensity of the two lines of each GFP variant. Each point represents an individual animal. Error bars represent ± standard errors of mean (SEM). n.s not significant; * p < 0.05; ** p < 0.01; *** p < 0.001; **** p < 0.0001. *n* = 10-11. (F-I) Representative images of the head of L4 animals expressing one of the GFP variants, (F) *eft-3* p::eGFP ( *mizSi29* ), (G) *eft-3* p::GFPnovo2 ( *mizSi31* ), (H) *eft-3* p::mNeonGreen ( *mizSi33* ), and (I) *eft-3* p::mStayGold ( *mizS35* ). The dashed circle represents the bleached area and region of interest used for quantification. Three bleaching events occurred between each time point shown. Scale bar: 5 μm. (J) Quantifications of the mean fluorescence intensity within the region of interest over time of the two lines of each GFP variant. Values represent mean ± SEM. *n* = 10. (K) Quantifications of the mean fluorescence intensity of mNeonGreen and mStaygold normalized to pre-bleaching signal intensities.

## Description

Since the initial discovery and purification of the green fluorescent protein (GFP) from the jellyfish *Aequorea victoria* by Shimomura et al. (1962), and the report of GFP as a tool to visualize neurons in *Caenorhabditis elegans* (Chalfie et al., 1994) , GFP has been invaluable for visualizing cells and proteins and monitoring gene expression across model systems. Efforts in protein engineering and screening over several decades have generated variants of GFP that are monomeric, and exhibit increased brightness and photostability. Some of these GFP variants are eGFP, GFPnovo2, mNeonGreen, and mStayGold. eGFP has a 100-fold increase in fluorescence intensity compared to the original GFP (Cormack et al., 1996; Yang et al., 1996) and GFPnovo2 which is derived from eGFP, is 3.3 times brighter than eGFP (Arakawa et al., 2008) . mNeonGreen is derived from a tetrameric yellow fluorescent protein, LanYFP which exhibits a high quantum yield and extinction coefficient, from *Branchiostoma lanceolatum* (Shaner et al., 2013) . mStayGold is derived from a highly bright and photostable dimeric green fluorescent protein, StayGold, from *Cytaeis uchidae* (Ando et al., 2023; Hirano et al., 2022) . Here, we examined the fluorescence intensity and photostability of eGFP, GFPnovo2, mNeonGreen, and mStayGold in the head region of *C. elegans* by expressing each fluorescent protein under the *eft-3* ubiquitous promoter.

For direct comparisons among GFP variants, we generated single copy knock-in animals that carry 1) *eft-3* p *::eGFP* ( *mizSi29* , *mizSi30* ), 2) *eft-3* p *::GFPnovo2* ( *mizSi31* , *mizSi32* ), 3) *eft-3* p::mNeonGreen ( *mizSi33* , *mizSi34* ) and 4) *eft-3* p::mStayGold ( *mizS35* , *mizSi36* ) near the *cxTi10882* MoSCI site on chromosome IV using the CRISPR/Cas9 genome editing technology. Using these knock-in strains, we examined the fluorescence intensity of the GFP variants in the heads of 4 ^th^ larval stage (L4) animals. We found that fluorescent signals of mStayGold were noticeably brighter than those of other GFPs ( Fig. 1A -1D). Consistently, the mean fluorescence intensity was highest in the mStayGold strains, followed by the mNeonGreen, GFPnovo2, and eGFP strains ( Fig. 1A -1E). The results show that mNeonGreen and mStayGold are significantly brighter than eGFP and GFPnovo2, which confirms previous works using cultured cells (Shaner et al., 2013; Ando et al., 2023; Hirano et al., 2022) , and using extrachromosomal arrays in *C. elegans* (Zhang et al., 2024) .

We then analyzed the photostability of each GFP variant in L4 animals by repeatedly bleaching an area of interest in the isthmus of the pharynx. Bleaching occurred nine times in a defined region using a high laser power (80%). Confocal images were taken once before the initial bleaching event (t=0) and following each bleaching event (t=1-9). Consistent with the original works using cultured cells (Ando et al., 2023; Hirano et al., 2022) , we found that mStayGold was the most photostable GFP variant, with cell bodies remaining visible within the bleached area even after photobleaching nine times (t=9) ( Fig. 1I, 1J). Fluorescent signals from cell bodies in the regions of interest in the eGFP, GFPnovo2, and mNeonGreen lines were barely visible following the nine bleaching events ( Fig. 1F -1H and 1J). We also noticed that mNeonGreen signal bleached faster than other fluorescent proteins, even though its initial brightness was substantially higher than that of eGFP and GFPnovo2 ( Fig. 1H -1K). This observation is consistent with recent work showing that mNeonGreen is less photostable than another monomeric StayGold variant, mBaoJin (Zhang et al., 2024) . It is important to consider that the harsh conditions of our photobleaching experiment are unlikely to resemble those of typical live imaging conditions. These results show that mStayGold can endure the harsh conditions of our photobleaching experiment, suggesting that it may hold advantages over eGFP, GFPnovo2, and mNeonGreen in experiments where photostability is an important factor, such as in time-lapse imaging.

**Limitations of the work**

Here, by using single-copy knock-in strains, we confirmed that the codon-optimized mStayGold is brighter and more photostable than the other tested GFP variants in *C. elegans* . While we did not compare mStayGold and other GFP variants when fused with endogenous proteins, the brightness of mStayGold is likely to be beneficial for labeling endogenous proteins with low expression levels or for visualizing microstructures such as cilia. The photostability of mStayGold may be beneficial for time-lapse imaging. While this work confirms the bright and photostable natures of mStaygold originally shown in cultured cells (Ando et al., 2023; Hirano et al., 2022) , we do not exclude the possibility that the codon-optimization we made to mStayGold increased its translation efficiency. Despite the many advantages of mStayGold, its use may be limited due to potential incompatibilities with some experimental techniques. Notably, there remains limited availability of antibodies to detect mStayGold, and hence mStayGold is not compatible with commonly used degron systems, such as the GFP-nanobody-based auxin-inducible degron (AID) system (Daniel et al., 2018; Wang et al., 2017) . Finally, the photostability of mStayGold may be disadvantageous for Fluorescence Recovery After Photobleaching (FRAP) experiments, where bleaching of fluorescent proteins in the region of interest is necessary to quantify recovery rates. We suggest mNeonGreen for FRAP experiments, as this work and others showed that mNeonGreen photobleached faster than monomeric versions of StayGold (Zhang et al., 2024) . Future studies may work to develop resources to improve the compatibility of the bright and photostable mStayGold with other experimental techniques for further applications.

## Methods

***General maintenance***

All *C. elegans* strains were generated from Bristol N2 wild-type strains and cultured on nematode growth medium (NGM) with OP50 *Escherichia coli* as described by Brenner (1974). All strains were kept at room temperature (22°C). General maintenance was conducted using stereomicroscopes (ZEISS Stemi 305 and ZEISS SteREO Discovery V8).

All strains used in this study are listed in the reagents section.

***Codon optimization of mStayGold***

A codon-optimized mStayGold with two synthetic introns was designed using the codon adapter tool ( https://worm.mpi-cbg.de/codons/cgi-bin/optimize.py ) (Redemann et al., 2011) and the GeneArt Codon Optimization tool. The DNA fragment was synthesized using GeneArt (ThermoFisher Scientific). The sequence of codon-optimized mStayGold is shown below. The small letters indicate synthetic introns.

ATGGTTTCAACGGGAGAAGAACTTTTCACAGGTGTCGTGCCATTCAAGTTCCAACTCAAGGGAACCATCAACGGAAAGTCGTTCACCGTTGAAGGAGAGGGAGAGGGAAATTCACATGAGGGATCACACAAGGGAAAATACGTGTGCACATCGGGAAAGCTCCCAATGTCATGGGCTGCTCTTGGAACATCATTCGGATACGGAATGAAGTACTACACGAAGTACCCGTCGGGACTCAAGgtaagtttaaacatatatatactaactaaccctgattatttaaattttcagAACTGGTTCCACGAAGTGATGCCAGAGGGATTCACATACGATCGACACATCCAGTACAAGGGAGATGGATCGATTCACGCTAAGCACCAGCATTTCATGAAGAACGGAACCTACCACAACATCGTCGAGTTCACCGGACAGGATTTCAAGgtaagtttaaacagttcggtactaactaaccatacatatttaaattttcagGAGAACTCGCCGGTCCTCACGGGAGATATGGATGTTTCACTCCCAAACGAGGTGCAGCACATTCCAATCGATGATGGTGTTGAGTGCACAGTGACCCTTCAATACCCACTCCTCTCGGATGAGTCAAAATGCGTTGAGGCCTACCAGAACACGATCATTAAGCCACTCCACAATCAGCCAGCTCCAGATGTTCCATTCCACTGGATTCGAAAGCAGTACACCCAGTCGAAGGATGATACAGAGGAACGTGATCACATCATCCAGTCAGAAACACTCGAAGCCCATCTCTGA

***Strain development***

The green fluorescent protein expression constructs were generated in the pSM plasmid which is a derivative of pPD49.26 (A. Fire). eGFP derived from pPD49.26 (A. Fire), GFPnovo2 (Hendi and Mizumoto, 2018) , mNeonGreen (Artan et al., 2021) , and a codon-optimized mStayGold were cloned into the *Kpn* I and *Eco* RI sites of the pSM vector. The 609 bp sequence of the *eft-3* promoter was cloned into the *Sph* I and *Asc* I sites. The single copy knock-in was conducted by using CRISPR/Cas9 genome editing technology near the cxTi10882 locus on chromosome IV. gRNA and the primer sequences with 80 bp homology arm sequences are listed below. These primers bind to the backbone sequences of the pSM vector and amplify *eft-3* p *::fluorescent protein::unc-54utr.* The CRISPR/Cas9 injection was conducted according to the previous work (Ghanta et al., 2020) . The screening of knock-in animals was conducted based on the dim and uniform expression of the green fluorescent signal under the fluorescence stereoscope (ZEISS SteREO Discovery V8).

gRNA: ACAAGTGTCGTTGACCCAGT

HDR primer forward: aagaaccctgattctgtcaagcctatgaagatttaaaaaaaattgggaagacccttagttccaaacaagtgtcgttgacATGACCATGATTACGCCAAGC

HDR primer reverse: aacattcctagctaaatgtaagttagcgaccaattttttagcaaccccattttatgacttttcagaatatcgcctactgGAAACGCGCGAGACGAAAGGG

***Laser scanning confocal microscopy***

Fluorescent proteins were imaged in live *C. elegans* using a Zeiss LSM800 Airyscan confocal microscope equipped with a 40× magnification oil objective lens. The same settings (laser power, exposure time, and gain) were used for each genotype. The same excitation wavelength (488 nm) and emission wavelength (509 nm) were used for each data set. We note that we did not use the optimal excitation wavelengths for mNeongreen (506 nm) and mStaygold (499 nm), and a better signal would be expected if the optimal excitation wavelengths were used. Live *C. elegans* were immobilized on a 2.5% agarose pad using a mixture of 0.225M 2,3-butanedione monoxime and 7.5 mM levamisole. L4.4-L4.6 stages of animals, defined by the morphology of the developing vulva (Mok et al. *,* 2015), were used for data collection.

***Fluorescence intensity***

Confocal microscopy was used to image the fluorescent signal from the head region and pharynx of the animals. 40 z-stack images with 0.58 μm intervals were used for each image. Representative images and images used for quantification are maximum projection images. The average fluorescence intensity of each image was measured using ImageJ.

***Photobleaching***

Photobleaching experiments were conducted using confocal microscopy. Bleaching occurred nine times using a high laser power (80%). Each bleaching duration was 20 seconds. Images were taken before bleaching (t=0) and after each bleaching (t=1-9) for a total of ten images. 15 Z-stacks at 0.50 μm intervals were used for each image. Representative images and images used for quantification are maximum projection images. The average fluorescence intensity within the bleached area was measured using ImageJ.

***Statistics***

Fluorescence intensity and photobleaching data were analyzed using Prism10. A one-way ANOVA method was used and corrected with post hoc Tukey's multiple comparisons tests between all genotypes to compare average fluorescence intensities. Error bars represent SEM. n.s. represents not significant. *, **, ***, and **** represent p-values <0.05, <0.01, <0.001, and <0.0001 respectively.

## Reagents

**Table:** 

**Strains**		
Strain	Genotype	Source
UJ3172	*mizSi29* [ *eft-3* p *::eGFP* ] IV	This study
UJ3173	*mizSi30* [ *eft-3* p *::eGFP* ] IV	This study
UJ3174	*mizSi31* [ *eft-3* p *::GFPnovo2* ] IV	This study
UJ3175	*mizSi32* [ *eft-3* p *::GFPnovo2* ] IV	This study
UJ3176	*mizSi33* [ *eft-3* p *::mNeonGreen* ] IV	This study
UJ3177	*mizSi34* [ *eft-3* p *::mNeonGreen* ] IV	This study
UJ3178	*mizSi35* [P *eft-3 ::mStayGod* ] IV	This study
UJ3179	*mizSi36* [P *eft-3 ::mStayGold* ] IV	This study

The plasmid containing *C. elegans* codon-optimized mStayGold will be available from Addgene.
